# Modular reconstruction and optimization of the *trans*-4-hydroxy-L-proline synthesis pathway in *Escherichia coli*

**DOI:** 10.1186/s12934-022-01884-4

**Published:** 2022-08-11

**Authors:** Zhenyu Zhang, Weike Su, Yunyun Bao, Qianqian Huang, Kai Ye, Pengfu Liu, Xiaohe Chu

**Affiliations:** 1 Collaborative Innovation Center of Yangtze River Delta Region Green Pharmaceuticals, Zhejiang University of Technology, Hangzhou, 310014 Zhejiang People’s Republic of China; 2 School of Pharmaceutical Sciences, Zhejiang University of Technology, Hangzhou, 310014 Zhejiang People’s Republic of China

**Keywords:** *Trans*-4-hydroxy-L-proline, *Escherichia coli*, Modular metabolic engineering, Metabolic balance

## Abstract

**Background:**

In recent years, there has been a growing demand for microbial production of *trans*-4-hydroxy-L-proline (*t*4Hyp), which is a value-added amino acid and has been widely used in the fields of medicine, food, and cosmetics. In this study, a multivariate modular metabolic engineering approach was used to remove the bottleneck in the synthesis pathway of *t*4Hyp.

**Results:**

*Escherichia coli t*4Hyp synthesis was performed using two modules: a α-ketoglutarate (α-KG) synthesis module (K module) and L-proline synthesis with hydroxylation module (H module). First, α-KG attrition was reduced, and then, L-proline consumption was inhibited. Subsequently, to improve the contribution to proline synthesis with hydroxylation, optimization of gene overexpression, promotor, copy number, and the fusion system was performed. Finally, optimization of the H and K modules was performed in combination to balance metabolic flow. Using the final module H1K4 in a shaking flask culture, 8.80 g/L *t*4Hyp was produced, which was threefold higher than that produced by the W0 strain.

**Conclusions:**

These strategies demonstrate that a microbial cell factory can be systematically optimized by modular engineering for efficient production of *t*4Hyp.

**Supplementary Information:**

The online version contains supplementary material available at 10.1186/s12934-022-01884-4.

## Background

*Trans*-4-hydroxy-L-proline (*t*4Hyp) is present in both animals and plants. As a component of collagen, a subcomponent that is complementary to acetylcholine esterase and elastin, *t*4Hyp is a nonessential amino acid in several secondary metabolites [1]. *T*4Hyp is essential to the thermodynamic stability of triple-helices [2]. As a value-added amino acid, *t*4Hyp has been widely used in the fields of medicine, food, and cosmetics [3]. For example, *t*4Hyp can be used to synthesize a large variety of chiral molecules, such as glutamate analogs and kainic acids, and natural products, such as lycoperdic acid, bulgecins, fully synthetic piperidines and pyrrolidines, benzodiazepines, puromycin analogs, baclofen and, notably, carbapenem antibiotics [4].

*Escherichia coli* (*E. coli*) does not synthesize *t*4Hyp because it lacks proline-4-hydroxylase (P4H), which catalyzes the conversion of L-proline to *t*4Hyp [5]. Nevertheless, *t*4Hyp has been successfully produced in *E. coli* [6], although product titers could be improved. To date, many efforts have been made to improve the production of *t*4Hyp. Shibasaki et al. utilized the P4H from *Dactylosporangium* sp. RH1 to produce *t*4Hyp in *E. coli*. With the addition of proline, *t*4Hyp reached a concentration of 41 g/L in 100 h [7]. In the same year, Shibasaki et al. constructed a plasmid carrying genes encoding feedback-resistant γ-glutamyl kinase (*proB*_*74*_) and *proA* and introduced it into *E. coli* W1485 ∆*putA*. The engineered strain directly produced 25 g/L *t*4Hyp in 96 h in the presence of glucose [6]. Recently, Wang et al. enhanced *t*4Hyp production by optimizing *p4h* gene codons in combination with mutagenesis and further optimized nutritional elements in a 5 L fermenter and achieved an output in fed batch mode of 25.4 g/L *t*4Hyp within 48 h [8]. Zhao et al. integrated the *Vitreoscilla hemoglobin* gene (*vgb*) into the chromosome of recombinant *E. coli* expressing the *p4h* gene from *Dactylosporangium* sp. RH1, and, using a shaking flask culture, obtained a 94.4% increase in *t*4Hyp production [9]. Wang et al. discovered a new P4H from *Alteromonas mediterranea* by genome mining. The engineered strain produced 45.83 g/L *t*4Hyp within 36 h [10]. Zhang et al. simultaneously deleting *sucC* and *sucD* genes, the engineered strain produced 4.81 g/L *t*4Hyp; this amount was 60% higher than the amount produced by the wild-type strain [11]. Jiang et al. enhanced L-proline biosynthesis by eliminating byproducts generated from L-proline, pyruvate, acetyl-CoA, isocitrate and optimizing the genes needed for L-proline biosynthesis. As a result, the engineered strain produced 4.82 g/L *t*4Hyp [12]. In order to enhance the activity and thermos-stability of P4H, a new P4H from the uncultured bacterium esnapd13 putative “lid” loop in combination with site-directed mutagenesis was performed. Finally, 12.9 g/L *t*4Hyp was obtained in a fed-batch fermentation [13]. Recently, Long et al. significantly enhancing production of *t*4Hyp through rare codon selected evolution, dynamic precursor modulation, and metabolic engineering. At last, 54.8 g/L *t*4Hyp was achieved in 60 h almost without L-proline remaining [14].

In contrast with the traditional metabolic engineering strategy, which may introduce a new bottleneck after each round of single precursor optimization, a modular metabolic engineering strategy can be used to optimize all precursors or pathways simultaneously, thus eliminating the limitations introduced by adding restrictions. Recent studies have shown that modular metabolic engineering can be used to balance the expression levels of genes to improve production. Darmawi et al. designed and constructed a modular biosynthetic pathway for L-tyrosine production in *E. coli* MG1655. According to the protein and metabolite measurements, optimization of the shikimate module and tyrosine module was performed. As a result of expressing two medium-copy-number, dual-operon plasmids, 2 g/L L-tyrosine was obtained at 80% of the theoretical yield [15]. Liu et al. performed modular engineering with *E. coli* to improve flavin production by dividing the RF operon and the bifunctional RF kinase/FAD synthetase into two separate modules and expressing the genes at different levels. Using this method, the titers of FAD and FMN produced during shake flask fermentation were as high as 324.1 mg/L and 171.6 mg/L, respectively [16].

**Fig. 1:**
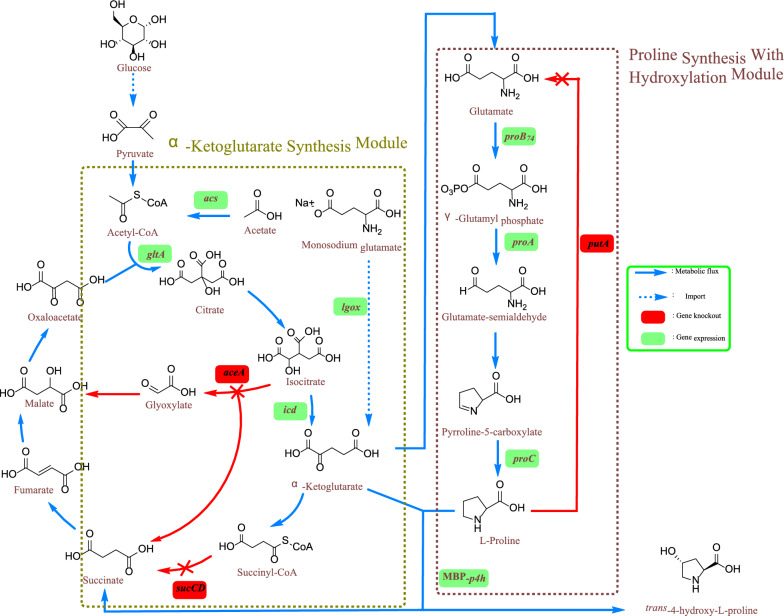
Engineered metabolic pathway for the production of *t*4Hyp in *E. coli. putA* (encoding proline dehydrogenase), *sucCD* (encoding ketoglutarate dehydrogenase complex), *aceA* (encoding isocitrate lyase), *proB*_*74*_ (encoding feedback resistant γ-glutamate kinase), *proA* (encoding glutamate-5-semialdehyde dehydrogenase), *proC* (encoding pyrroline-5-carboxylate reductase), MBP-*p4h* (encoding proline-4-hydroxylase from *Dactylosporangium* sp with MBP), *acs* (encoding acetyl-CoA synthetase), *gltA* (encoding citrate synthase), *icd* (encoding isocitrate dehydrogenase), *lgox* (encoding L-glutamate oxidase)

In this study, we aimed to improve the availability and balance of precursors to satisfy *t*4Hyp manufacturing requirements. The key genes in central metabolic and proline synthesis pathways were examined. At the same time, we focused on the hydroxylation capacity of the engineered strain; that is, we optimized P4H expression. To address the imbalance in the expression levels of precursors, a modular metabolic engineering strategy was introduced to further optimize the production of *t*4Hyp. The *t*4Hyp biosynthesis pathway was partitioned into two modules: a α-KG module and L-proline with hydroxylation module. At the level of transcriptional regulation, manipulation of various promoters was performed to balance gene expression levels. In addition, due to the introduction of L-glutamate oxidase (LGOX), which converts L-glutamate to α-KG, *t*4Hyp synthesis from glucose and monosodium glutamate (MSG) was improved. To our knowledge, this study is the first to investigate the effects of a modular metabolic engineering strategy on *t*4Hyp production while taking advantage of MSG. The proposed *t*4Hyp pathway in *E. coli* is shown in Fig. 1.

## Results and discussion

### Reconstruction of proline and central metabolic pathway

The main limiting factors, which is used as the substrate for *t*4Hyp production, were α-KG and proline accumulation [17]. To solve this problem, the flux of carbon channeled from glucose into the tricarboxylic acid cycle (TCA) and proline needed to be improved. To prevent consumption of the proline pool, the *putA* gene (encoding proline dehydrogenase) was deleted to block the conversion of proline to corresponding glutamate [18, 19].

Following a procedure cited in the literature [6], the P1 plasmid was constructed and transformed into W3110 and R1-R8 strains, respectively. As shown in Table 1, the W1 (Δ*putA*) produced 5.90 g/L *t*4Hyp, which was twice that produced by the W0 strain. This result was consistent with the fact that proline is an important precursor for *t*4Hyp production and that deletion of *putA* leads to increased proline uptake.

**Table 1:** Physiological parameters of recombinant *E. coli* W3110 strains

	W0	W1	W2	W3	W4	W5	W6	W7	W8
Growth rate (g cdw/L/h)	0.067 ± 0.005	0.077 ± 0.001	0.059 ± 0.002	0.050 ± 0.004	0.051 ± 0.001	0.052 ± 0.002	0.057 ± 0.004	0.055 ± 0.003	0.059 ± 0.003
Final biomass (g cdw/L)	4.84 ± 0.35	5.55 ± 0.06	4.24 ± 0.11	3.58 ± 0.27	3.65 ± 0.10	3.72 ± 0.15	4.09 ± 0.28	3.99 ± 0.21	4.25 ± 0.22
C *t*4Hyp (g/L)	2.94 ± 0.27	5.90 ± 0.29	3.72 ± 0.27	1.80 ± 0.03	3.88 ± 0.29	5.60 ± 0.14	2.51 ± 0.06	7.65 ± 0.20	7.89 ± 0.16
Y *t*4Hyp (g *t*4Hyp/g cdw)	0.61 ± 0.05	1.07 ± 0.06	0.88 ± 0.04	0.51 ± 0.02	1.06 ± 0.06	1.51 ± 0.09	0.62 ± 0.05	1.92 ± 0.05	1.86 ± 0.06
Y *t*4Hyp (g *t*4Hyp/g glc)	0.07 ± 0.01	0.12 ± 0.01	0.11 ± 0.00	0.06 ± 0.00	0.13 ± 0.01	0.16 ± 0.00	0.08 ± 0.00	0.21 ± 0.00	0.22 ± 0.00
r *t*4Hyp (g *t*4Hyp/L/h)	0.041 ± 0.004	0.082 ± 0.004	0.052 ± 0.004	0.025 ± 0.000	0.054 ± 0.004	0.078 ± 0.002	0.035 ± 0.001	0.106 ± 0.002	0.110 ± 0.002

To improve α-KG accumulation, we focused mainly on the central metabolic pathways by altering α-KG and isocitrate metabolism to increase the availability of α-KG, and the carbon flux was redirected from α-KG to *t*4Hyp [20–22]. Recombinant *E. coli* strains were constructed by deletion of α-ketoglutarate dehydrogenase (sucA) and/or isocitrate lyase (aceA). The *t*4Hyp concentration of W2 (Δ*sucA*) was increased to 3.72 g/L, which was 27% higher than that of W0. While deletion of *aceA* had a negative effect. Critically, the *t*4Hyp concentration only 1.80 g/L in W3 (Δ*aceA*), 39% lower than that of W0. The *t*4Hyp yield on glucose was also decreased to 0.06 g/g in W3 (Δ*aceA*), 14% lower than that of W0. Furthermore, the W2 (Δ*sucA*) strain exhibited a lower growth rate, final biomass and glucose consumption rate as compared to W0. These outcomes may have been due to the deletion of *sucA,* which caused the breakdown of the TCA cycle. The supply of oxaloacetate and succinate might rely on the glyoxylate cycle [23]. The deletion of *aceA* in *E. coli* severely impaired the cell growth. As compared to W2 (Δ*sucA*), the greater severity of the *aceA* deletion and the higher functional significance of *aceA* in the TCA cycle also became obvious from the lower growth rate and the lower biomass. The highest metabolic pressure is for cell growth dependent on the proline hydroxylation, that is, *t*4Hyp synthesis in fermentation [24]. It is likely that the deletion of *aceA* have negative effects on metabolism, and that one consequence is inefficient production of *t*4Hyp. This may be due to the fact that PEP carboxylation is the only anaplerotic route for oxaloacetate replenishment in glyoxylate shunt-deficient *E. coli* [25]. The strain deletion of both sucA and aceA, in fact, unable to grow in minimal medium. The expression of P4H can restored cell growth and proline hydroxylation as an alternative bypass to restore a TCA cycle. Furthermore, the growth of the Δ*sucA*Δ*aceA* mutant strain is coupled to proline hydroxylation. However, despite the presence of proline hydroxylation, the reduction of sucCoA and the glyoxylate shunt-deficient in the Δ*sucA*Δ*aceA* strain imposed a heavy stress on cell growth as emphasized by the lower growth rate and final biomass concentration obtained as compared to the W0 [24]. Strain W4 (Δ*sucA*Δ*aceA*, 3.88 g/L), which was no obvious improvement compare with W2 (Δ*sucA*, 3.72 g/L), but 115% higher than that produced by strain in W3 (Δ*aceA*, 1.80 g/L). The results showed that although a metabolic burden was imposed by heterologous *t*4Hyp synthesis and despite the reduction in energy and precursors available for biomass formation, the simultaneous elimination of *sucA* and *aceA* increased the flux through P4H, resulting in an increased *t*4Hyp titer.

To investigate the effects of modifying genes in combination on the central metabolic and proline degradation pathways, a knockout assay was performed. Deletion of both *putA* and *sucA* exerted additive effects on *t*4Hyp production, and 5.60 g/L *t*4Hyp was obtained, a titer 51% higher than that of W2 (Δ*sucA*). In the W6 (Δ*putA*Δ*aceA*) strain, the rate of *t*4Hyp formation was much faster than that in the W3 (Δ*aceA*) strain. Compared to that of the W3 strain, the final titer was increased by 39%, from 1.80 g/L to 2.51 g/L. In the triple-deletion mutant W7 (Δ*putA*Δ*sucA*Δ*aceA*) strain, after 72 h of cultivation in a flask, 7.65 g/L *t*4Hyp was detected in the culture broth, which was a significant increase in *t*4Hyp formation; this titer was 97% higher than that of the W4 (Δ*sucA*Δ*aceA*) strain. These results indicated that the combination of modified genes in the central metabolic and proline degradation pathways exerted a synergetic effect to increase *t*4Hyp production in *E. coli*. Deletion of the *sucA* gene may have resulted in an adverse effect on cell growth due to the reduction in sucCoA level. The W8 (Δ*putA*Δ*sucCD*Δ*aceA*) strain was constructed for further evaluation. Compared with those in the W7 strain, the final biomass and growth rate were both improved in the W8 strain. For *t*4Hyp production, the titer and yield were increased to 7.89 g/L and 0.22 g/g glucose, respectively. These results indicated that the reduction of sucCoA disadvantaged cell growth and thus had an effect on *t*4Hyp production. Considering the *t*4Hyp production ability in *E. coli*, we selected the Δ*putA*Δ*sucCD*Δ*aceA* strain for use in further experiments.

### Investigating *trans*-4-hydroxy-L-proline production by overexpressing genes in the proline biosynthesis pathway

**Fig. 2:**
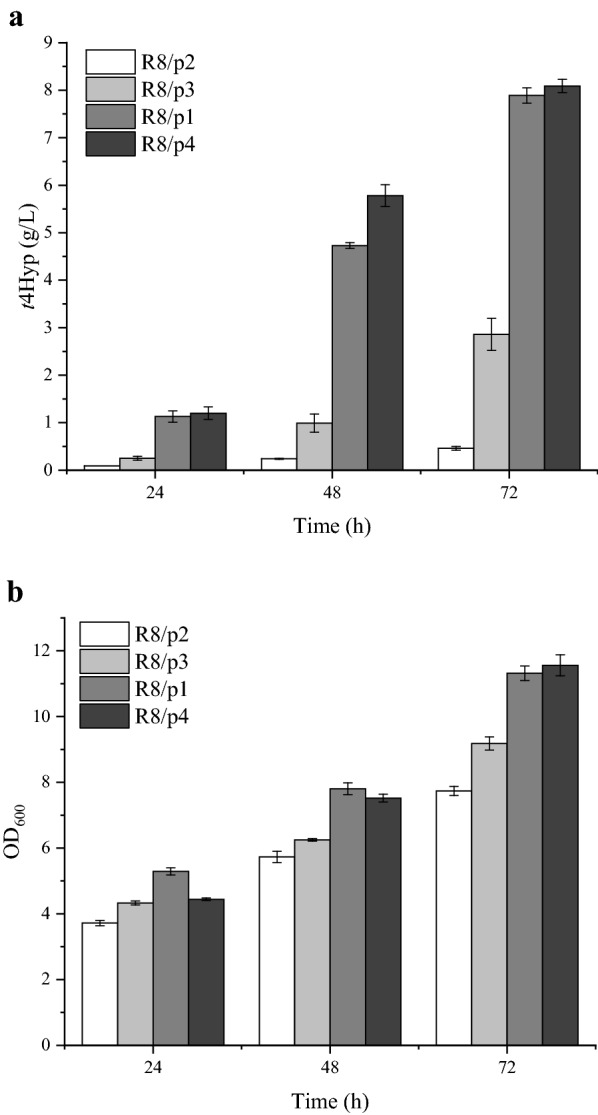
Effects of the overexpression of proline biosynthesis pathway genes. **a** *t*4Hyp titers. **b** Cell concentration. (R8: W3110 ∆*putA*∆*sucCD*∆*aceA*; p2: pET24a, P*trp*::*p4h*; p3: pET24a, P*trp*::*p4h-proB*_*74*;_ p1: pET24a, P*trp*::*p4h*-*proB*_*74*_-*proA*; p4: pET24a, P*trp*::*p4h-proB*_*74*_*-proA-proC*)

One approach to enhance *t*4Hyp production involved increasing the expression of upstream intermediates in the proline biosynthesis pathway. In *E. coli*, glutamate is the primary precursor of proline synthesis [26]. Proline biosynthesis from glutamate is realized via three enzymatic reactions that are catalyzed by γ-glutamyl kinase (ProB), glutamate-γ-semialdehyde dehydrogenase (ProA), and Δ^1^-pyrroline-5-carboxylate reductase (ProC) [27, 28]. Thus, the expression of three genes (*proB*_*74*,_ *proA*, and *proC*) related to *E. coli* proline biosynthesis was moderated to enhance proline synthesis [26]. Because proline produces a feedback inhibition effect on ProB, which is the rate-limiting step in proline biosynthesis, feedback-inhibition resistant ProB_74_ was used in this study [29, 30]. Gene *p4h* from *Dactylosporangium* sp. was codon optimized and expressed in *E. coli* to enable *t*4Hyp production. As shown in Fig. 2, small amounts of *t*4Hyp and biomass were produced; when R8/p2 was cultivated, the titer and OD_600_ were only 0.46 g/L and 7.74, respectively. However, when the *proB*_*74*_ gene together with the *p4h* gene were overexpressed in *E. coli*, R8/p3 was obtained. The expression of *proB*_*74*_ clearly increased the titer and biomass, and 2.86 g/L and OD_600_ 9.18 were obtained, which were 6.2- and 1.2-fold those of R8/p2, respectively.

As an alternative way to enhance the flux to proline, we examined the effects of overexpressing *proA*. As expected, increasing the expression of the *proA* gene further increased the production titer to 7.89 g/L, which was 2.8-fold higher than that of R8/p3. This result indicates that coexpressing *proB*_*74*_ and *proA* resulted in a synergistic effect on *t*4Hyp production. In addition, the OD_600_ of W8 was 11.32, which was 23% higher than that of R8/p3.

Based on the promising results obtained using the W8 strain for the production of *t*4Hyp, further metabolic engineering was performed to increase *t*4Hyp production. In this experiment, the co-overexpression of *p4h*, *proB*_*74*_, *proA*, and *proC* resulted in a *t*4Hyp titer of 8.09 g/L, which was 17.6-fold that of R8/p2. This result confirmed that *proC* overexpression enabled further increase in *E. coli t*4Hyp production, although the increase was slight. Two reasons may explain this outcome: either the accumulation of proline was sufficient (6.78 g/L), or P4H activity was restricted. The *t*4Hyp-producing cells, especially the high-level producers, showed a significant titer and yield production level, suggesting that the metabolically engineered proline production pathway competed with the arginine production pathway while allowing higher flux to be directed toward *t*4Hyp. These results also directly suggest that the proline route is a promising alternative to replenish the previously consumed α-KG during the production of proline-derived products. Overall, our results demonstrated that overexpression of three genes (*proB*_*74*_, *proA*, and *proC*) improved total *t*4Hyp production through their enhanced effect on proline synthesis.

### Optimization of proline-4-hydroxylase expression by employing different expression systems

During *t*4Hyp synthesis in *E. coli*, α-KG and proline conversion into *t*4Hyp is thought to be a rate-limiting step that is mainly driven by P4H. By enhancing the expression of P4H, α-KG and proline can be more efficiently consumed in the synthesis of *t*4Hyp. In view of this possibility, the *p4h* gene was ligated into the pACYC-Duet-1, pET24a, pKK223-3, pGEX-6P-1, pMAL-C2-X, pET20b, pET39b, pET43.1a, and pET48b expression vectors and expressed in *E. coli* BL21 (DE3), and these strains were called B1, B2, B3, B4, B5, B6, B7, B8, and B9 strains, respectively. Thus, different plasmid copy numbers, promoters, and fusion protein tags were chosen to improve the expression of P4H. All constructs were evaluated on the basis of P4H activity in recombinant whole cells.

**Fig. 3:**
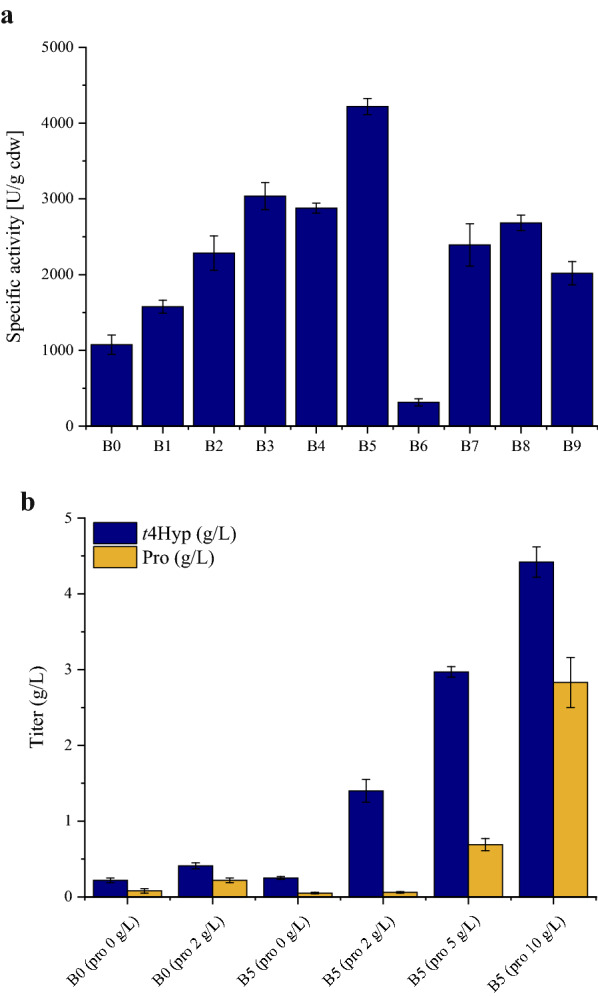
Effect of vectors on P4H activity and *t*4Hyp production. **a** The specific activity of the B0-B9 strains. **b** The *t*4Hyp production of the B0 and B5 strains. (B0: BL21 (DE3), p2: pET24a, P*trp*::*p4h*; B1: BL21 (DE3), p5: pACYC-Duet-1, P*trp*::*p4h*; B2: BL21 (DE3), p6: pET24a, *p4h*; B3: BL21 (DE3), p7: pKK223, *p4h*; B4: BL21 (DE3), p8: pGEX-6P-1, *p4h*; B5: BL21 (DE3), p9: pMAL-C2-X, *p4h*; B6: BL21 (DE3), p10: pET20b, *p4h*; B7: BL21 (DE3), p11: pET39b, *p4h*; B8: BL21 (DE3), p12: pET43.1a, *p4h*; B9: BL21 (DE3), p13: pET48b, *p4h*)

With L-proline, α-KG, and Fe^2+^ as substrates, different levels of catalytic activity were observed when recombinant *E. coli* BL21 (DE3) expressed different expression vectors. As shown in Fig. 3a, the P4H catalytic activities of B0 (1076 U g^−1^ cdw) and B1 (1577 U g^−1^ cdw) were observed. Plasmid of p5, with a low plasmid copy number, showed higher P4H catalytic activity. In strain B2, with the T7 promoter, and B3, with the tac promoter, the P4H activities were 2284 U g^−1^ cdw and 3036 U g^−1^ cdw, respectively. Compared with those in the B0 (trp promoter) strain, the P4H activities were improved approximately 2.1-fold and 2.8-fold in B2 and B3, respectively. In addition to the effect of the pelB signal peptide (314 U g^−1^ cdw), the activities of P4H were significantly increased with the use of a fusion protein tag; that is the activity levels were 2–3 times those of B0. These results suggested that the expression of P4H with fused protein tags increased the ability of P4H to convert proline and α-KG to *t*4Hyp. Among all the recombinant *E. coli* strains*,* the highest P4H activity was measured in the *E. coli* BL21 (DE3) strain expressing the p9 plasmid. The P4H activity reached 4218 U g^−1^ cdw. MBP is a maltose-binding protein with excellent ability that poor expression of proteins are often expressed better after fusion with MBP [31, 32].

In addition to examining the influence of expression vectors on P4H activity levels, *t*4Hyp production needed to be verified, and to this end, the B5 strain and B0 strain with different concentrations of proline were cultivated in shaking flasks. As shown in Fig. 3b, the titers of *t*4Hyp were 0.25 g/L and 1.4 g/L, which were increased to 13.6% and 241% in the B5 strain compared to the B0 strain supplemented with 0 and 2 g/L proline, respectively. When the proline concentration in the fermentation medium was increased to 5 g/L proline, the titer of *t*4Hyp in strain B5 reached 2.97 g/L, the residual proline was 0.69 g/L, and 69% proline was converted to *t*4Hyp. Furthermore, *t*4Hyp production was achieved at 4.42 g/L when 10 g/L proline was added, the residual proline was 2.83 g/L, and 62% proline was converted to *t*4Hyp.

In view of their excellent catalytic activity of L-proline, the *p4h*, *proB*_*74*_, *proA*, and *proC* genes were integrated into pMAL-C2-X to generate the p14 plasmid, and the *malE* gene (encoding MBP) was integrated into p4 to generate the p15 plasmid. The engineered strain harboring p15 showed greater cell growth than its counterpart, the engineered strain harboring p14. Finally, 8.23 g/L *t*4Hyp was synthesized in R8/p15 in a shake flask. In R8/p14, the titer of *t*4Hyp was only 3.65 g/L, which was 55% lower than that of R8/p4. This result may be ascribed to the strong promoter introduced into the plasmid, which may have prevented balanced expression of genes in the pathway; however, it is unclear whether the *t*4Hyp yield was slightly improved with MBP expression under a weaker promoter. Clearly, the overall cooperative regulation of pathways should be examined.

### Separation of the *trans*-4-hydroxy-L-proline synthesis pathway into two modules

Common metabolic engineering strategies were applied to improve *t*4Hyp production by knocking out competing pathways to increase carbon flux toward proline and α-KG, overexpressing bottleneck enzymes, and optimizing expression vectors to increase P4H activity. Based on the results, other rate-limiting factors need to be assessed. To circumvent current limitations, we took a modular metabolic engineering approach to optimize the metabolic balance between hydroxylation, the proline biosynthesis pathway and the α-KG biosynthesis pathway.

In our work, on the basis of the biosynthetic pathway of *t*4Hyp and considering metabolic burden and plasmid stability, the entire pathway was divided into two modules: (i) The H module was the hydroxylation and proline biosynthesis module. P4H and genes for synthesizing proline were placed in this module to modulate the level of hydroxylation and the amount of proline. (ii) The K module was the α-KG biosynthesis module, in which the amount of α-KG was modulated. Using a Gibson assembly [33], the H and K modules were successfully expressed by two compatible vectors.

### Engineering the H module to produce *trans*-4-hydroxy-L-proline

**Fig. 4:**
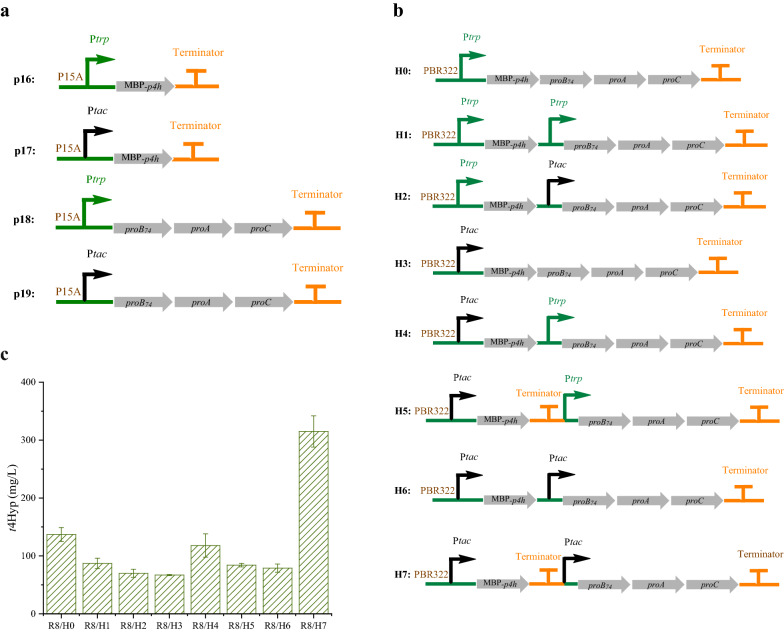
Transcriptional fine tuning of the H module (performed in 24 deep wells). **a** Description of the plasmids in which genes were incorporated for proline hydroxylation and biosynthesis. **b** Schematic of the H module design. **c** Production of *t*4Hyp in the engineered strains. (R8: W3110 ∆*putA*∆*sucCD*∆*aceA*)

As shown in Fig. 4a, to achieve the optimal distribution of carbon flux in the H module, *MH* (MBP-*p4h*) and *B*_*74*_*AC* (*proB*_*74*_*, proA*, and *proC*) were overexpressed at different expression strengths: a low level (under the trp promoter) and a high level (under the tac promoter). When the expression of *MH* and *B*_*74*_*AC* was low (with the trp promoter), the highest concentrations of *t*4Hyp and proline were 91 mg/L and 1754 mg/L, which were 2.2-fold and 1.4-fold higher than that the concentrations obtained when the expression was high. Subsequently, the H1 to H7 plasmids were transformed into strain R8 to examine their *t*4Hyp production ability (Fig. 4b). As shown in Fig. 4c, compared with the lowest expression level, which was in the R8/H0 strain (137 mg/L), maintaining the *MH* expression at a high level with a T7 terminator and high *B*_*74*_*AC* expression led to a twofold increase in the R8/H7 strain production of *t*4Hyp, which was as high as 315 mg/L. In the other strains, the titer of *t*4Hyp was lower than that of R8/H0, and there were no obvious differences. These results were similar to previous results: higher *t*4Hyp synthesis was observed when *MH* and *B*_*74*_*AC* were expressed under the trp promoter at a low transcription level. The only exception was in the strain expressing both *MH* and *B*_*74*_*AC* under the tac promoter at a high transcription level and both with the T7 terminator, which showed the highest *t*4Hyp synthesis ability. This result may be ascribed to the first tac promoter strongly leading to the read-through of the *B*_*74*_*AC* gene, affecting the metabolic balance. To prevent the read-through of subsequent genes, the T7 terminator was added after the *MH* gene, improving the expression and metabolic balance of *MH* and *B*_*74*_*AC*. Thus, the synthesis of *t*4Hyp was increased.

### Engineering the K module to produce α-ketoglutarate

**Fig. 5:**
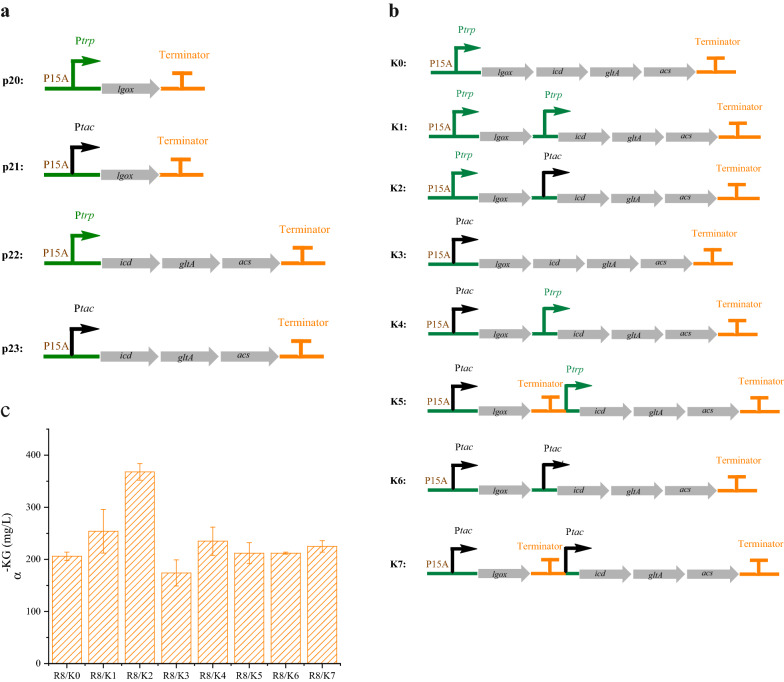
Transcriptional fine tuning of the K module (performed in 24 deep wells). **a** Description of the plasmids in which the genes for α-KG biosynthesis were incorporated. **b** Schematic of the K module design. **c** Production of α-KG in the engineered strains. (R8: W3110 ∆*putA*∆*sucCD*∆*aceA*)

Enhancing the supply of precursors is a key strategy to increase the flux toward *t*4Hyp production. Thus, to enhance α-KG biosynthesis, the K module was built to supplement the capacity of α-KG production from MSG and glucose via the genes *lgox* (encoding L-glutamate oxidase), *icd* (encoding isocitrate dehydrogenase), *gltA* (encoding citrate synthase), and *acs* (encoding acetyl-CoA synthetase). Similar to the previously constructed modules, *L* (*lgox*) and *IGa* (*icd*, *gltA*, and *acs*) were overexpressed to different degrees (Fig. 5a). For overexpression of *L*, the production of α-KG under the trp promoter was 1.4-fold greater, up to 128 mg/L higher than that realized with the tac promoter and 1 g/L MSG. A total of 92 mg/L α-KG was achieved when *IGa* were expressed under the trp promoter, but this was not significantly different than that expressed under the tac promoter. Subsequently, the K0-K7 engineering plasmids were transformed into strain R8 (Fig. 5b). The production of α-KG was then detected. As shown in Fig. 5c, with decreased expression of the upstream genes under the control of the trp promoter and increased expression of the downstream genes under the control of the tac promoter, the highest α-KG production, 368 mg/L, was achieved in strain R8/K2. Controlling *L* and *IGa* expression at a high level by using only one tac promoter led to the lowest titer of α-KG, 174 mg/L. All the other strains showed no significant difference in the production of α-KG. These results were similar to previous results: low transcription of the *L* gene promoted the conversion of MSG to α-KG in the medium, while a relatively high transcription level was not conducive to the synthesis of α-KG from the *L* gene or IGa genes. It is possible that the coexpression of the *L* gene and *IGa* genes in the R8/K2 strain was in an equilibrium state, thus enhancing the synthesis of α-KG.

### Balancing gene expression levels in two modules for enhancing *trans*-4-hydroxy-L-proline production from glucose and monosodium glutamate

**Fig. 6:**
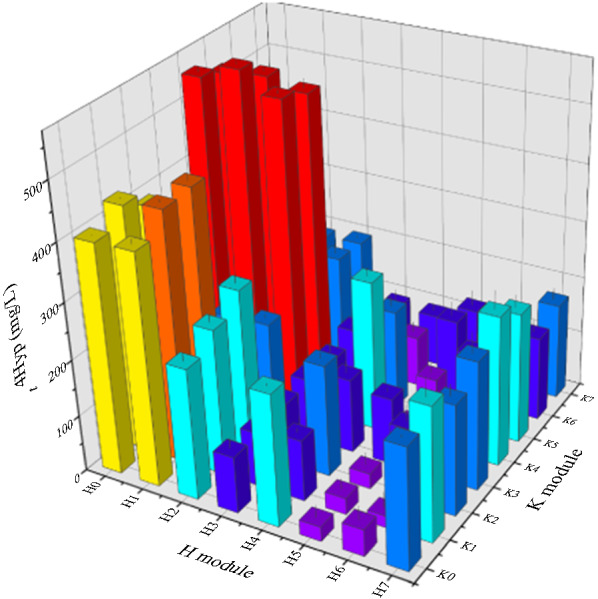
Optimization of *t*4Hyp production by balancing the two synthesis modules (performed in 24 deep wells)

With *t*4Hyp formation and the α-KG generation modules optimized separately, we next sought to balance the gene expression levels within the entire pathway. To perform this optimization, we combined the H and K modules to change the expression level of all genes. Thus, 64 combinatorial modules were generated (Fig. 6). Compatible plasmids were used to realize the strategy of producing a high copy number (pBR322 origin) and a low copy number (p15A origin) [34].

Interestingly, from the perspective of the respective titers of the two modules, the best result was not realized with either the combination of the highest titer in the H module with the highest titer in the K module or the combination of the lowest titer in the H module with the lowest titer in the K module; the best result was obtained through the combination of a medium titer in the H module with a medium titer in the K module. From the perspective of the transcription level of the two modules, the best result was obtained when both modules exhibited intermediate transcription levels. Finally, the results showed that the synthesis of *t*4Hyp could be best improved when the combination of H1K4 modules was optimized; that is, it was best when the expression of the *MH* and *B*_*74*_*AC* genes was relatively low or moderate, while the expression of *L* and *IGa* genes was relatively high and moderate, respectively. The *t*4Hyp titer of strain R8 with H1K4 was 575 mg/L, which was 320%, 82.5% and 375% higher than that of H0 (137 mg/L), H7 (315 mg/L), and p24 (121 mg/L), respectively. Moreover, the *t*4Hyp titers of H0K4 (550 mg/L), H1K4 (575 mg/L), and H2K4 (541 mg/L) were significantly improved compared with those of H0, indicating that overexpression of *MH* at a relatively low level, *L* at a relatively high level and *IGa* at a relatively moderate level significantly improved the production of *t*4Hyp in *E. coli*.

To verify the ability of the H1K4 module to produce *t*4Hypthe fermentation of strain R8 with H1K4 was performed in a shake flask after the addition of different concentrations of MSG. Similar to the results of recombinant cells screened in 24-deep-well plate cultures, H1K4 cells, *t*4Hyp was overproduced in the medium. With the addition of 1 g/L MSG, the *t*4Hyp titer of H1K4 was 8.60 g/L. With the addition of 5 g/L MSG, the highest *t*4Hyp titer of H1K4, 8.80 g/L, was reached, and the yield of glucose was approximately 0.24 g/g glucose, and these were 6.9% and 4.3% higher than those of H0, respectively. The titer and yield of *t*4Hyp were threefold and 3.4-fold higher than those of the W0 strain, respectively. However, when the addition of MSG was increased to 10 g/L, the *t*4Hyp titer of H1K4 was decreased to 8.32 g/L. This outcome may have been due to excessive MSG or the excessive conversion of α-KG from MSG, affecting the balance of metabolic flow or cell growth. In addition, within the range of 1–5 g/L MSG, the biomass of H1K4 was directly proportional to the concentration of MSG. Finally, the highest OD_600_ was 12.51. These results indicated that modular metabolic engineering used to modulate the flux of the important precursors of proline and α-KG and the rate of hydroxylation is a promising strategy to improve *t*4Hyp production. Differences in product synthesis were observed with the same genes at different expression levels, which also indicated the need to regulate the balance of expression levels among multiple genes.

In this research, we are focused on improving the production of *t*4Hyp. P4H is responsible for converting L-proline and α-KG to *t*4Hyp, which is crucial factor to improve the production of *t*4Hyp. It was reported that the evolution of P4H was effective to enhance hydroxylase activity and hydroxylation efficiency by genome mining or site-directed mutagenesis [7, 10]. The regulation of proline availability was also need to be proposed. To improve the hydroxylation efficiency, the regulation of protein level between the feedback-resistant γ-glutamyl kinase and hydroxylase by optimizing RBS was performed. The optimal engineered strain produces up to 21.72 g/L *t*4Hyp [11]. In addition, CRISPR interference may be an effective technique to down-regulation of target genes. It will be helpful to simultaneously increase target production and biomass when the technique is applied to modify the genes in the metabolic pathway [35, 36].

## Conclusions

In this study, the relationships between the proline degradation pathway and central metabolic pathway on *t*4Hyp production were investigated. Then, the genes in the proline biosynthesis pathway were overexpressed to increase *t*4Hyp production. To improve the efficiency of hydroxylation, optimization of the plasmid copy number, promoters, and fusion tags was performed. Finally, a modular metabolic engineering strategy was adopted to balance the expression levels of module genes by varying promoter strength. To our knowledge, this is the first report describing the modular metabolic engineering production of *t*4Hyp, which led to the highest titer reported for shake flask cultures.

## Materials and methods

### Media and growth conditions

All the strains were first precultured in LB medium (10 g/L peptone, 5 g/L yeast extract, and 10 g/L NaCl). The main culture was carried out using nutrient-rich medium (22 g/L glucose, 10 g/L (NH_4_)_2_SO_4_, 8 g/L peptone, 2 g/L NaCl, 1 g/L KH_2_PO_4_, 0.5 g/L MgSO_4_·7H_2_O, and 0.278 g/L FeSO_4_). During the main culture, the pH was adjusted to 7.0 by NaOH three times every 24 h, and additional glucose was added when the initial glucose was completely consumed. Recombinant *E. coli* BL21 (DE3) strains were grown in TB medium (4 ml/L glycerol, 24 g/L yeast extract, 20 g/L tryptone, 72 mM K_2_HPO_4_, and 17 mM KH_2_PO_4_). Ampicillin (100 μg/mL), kanamycin (50 μg/mL), and chloramphenicol (25 μg/mL) were added when necessary. Expression of genes in T7 or tac promoter constructs was induced by the addition of 0.2 mM isopropyl-β-D-thiogalactopyranoside (IPTG). The main cultures were routinely incubated in 250 mL baffled Erlenmeyer flasks or 24-deep-well plates at 34 °C, 220 rpm, and 72 h. The initial pH was approximately 7.0. The main culture was carried out in triplicate.

### Construction of plasmids and strains

All strains and plasmids used in this study are listed in Table 2. The primers used in this study are listed in Additional file 1. *E. coli* DH5α was used as the host for plasmid construction. *E. coli* BL21 (DE3) was used as the host for P4H expression. *E. coli* W3110 was used as a template and host for *t*4Hyp production.

**Table 2:** Strains and plasmids used in this study

Strains and plasmids	Relevant genotype or description	Source or reference
Strains		
W3110	Wild type *E. coli*	Laboratory stock
DH5α	Host cells for plasmids amplification	Laboratory stock
BL21 (DE3)	Host cells for plasmids expression	Laboratory stock
R1	W3110 ∆*putA*	[6]
R2	W3110 ∆*sucA*	This study
R3	W3110 ∆*aceA*	This study
R4	W3110 ∆*sucA*∆*aceA*	This study
R5	W3110 ∆*putA*∆*sucA*	This study
R6	W3110 ∆*putA*∆*aceA*	This study
R7	W3110 ∆*putA*∆*sucA*∆*aceA*	[24]
R8	W3110 ∆*putA*∆*sucCD*∆*aceA*	This study
W0	W3110 harboring p1	This study
W1	R1 harboring p1	This study
W2	R2 harboring p1	This study
W3	R3 harboring p1	This study
W4	R4 harboring p1	This study
W5	R5 harboring p1	This study
W6	R6 harboring p1	This study
W7	R7 harboring p1	This study
W8	R8 harboring p1	This study
R8/p2	R8 harboring p2	This study
R8/p3	R8 harboring p3	This study
R8/p4	R8 harboring p4	This study
B0	BL21 (DE3) harboring p2	This study
B1	BL21 (DE3) harboring p5	This study
B2	BL21 (DE3) harboring p6	This study
B3	BL21 (DE3) harboring p7	This study
B4	BL21 (DE3) harboring p8	This study
B5	BL21 (DE3) harboring p9	This study
B6	BL21(DE3) harboring p10	This study
B7	BL21 (DE3) harboring p11	This study
B8	BL21 (DE3) harboring p12	This study
B9	BL21 (DE3) harboring p13	This study
R8/p14	R8 harboring p14	This study
R8/p15	R8 harboring p15 (H0)	This study
R8/p16	R8 harboring p16	This study
R8/p17	R8 harboring p17	This study
R8/p18	R8 harboring p18	This study
R8/p19	R8 harboring p19	This study
R8/H1	R8 harboring H1	This study
R8/H2	R8 harboring H2	This study
R8/H3	R8 harboring H3	This study
R8/H4	R8 harboring H4	This study
R8/H5	R8 harboring H5	This study
R8/H6	R8 harboring H6	This study
R8/H7	R8 harboring H7	This study
R8/p20	R8 harboring p20	This study
R8/p21	R8 harboring p21	This study
R8/p22	R8 harboring p22	This study
R8/p23	R8 harboring p23	This study
R8/p24	R8 harboring p24	This study
R8/K0	R8 harboring K0	This study
R8/K1	R8 harboring K1	This study
R8/K2	R8 harboring K2	This study
R8/K3	R8 harboring K3	This study
R8/K4	R8 harboring K4	This study
R8/K5	R8 harboring K5	This study
R8/K6	R8 harboring K6	This study
R8/K7	R8 harboring K7	This study
Plasmids		
pKD46	Amp^R^ plasmid with temperature-sensitive replication and arabinose induction of λ-red recombinase	Laboratory stock
pCP20	Amp^R^ and Cm^R^ plasmid with temperature-sensitive replication and thermal induction of FLP synthesis	Laboratory stock
pET24a	High copy number vector, ColE1 ori, T7 lac promoter, Km^R^	Laboratory stock
pMAL-C2-X	*malE* gene encoding MBP fusion protein, tac promoter	Laboratory stock
pGEX-6P-1	GST fusion protein, tac promoter	Laboratory stock
pKK223-3	Amp^R^, tac promoter	Laboratory stock
pACYC-Duet-1	two multiple cloning sites, T7 lac promoter, P15A origin	Laboratory stock
pET20b	Bacterial vector for expressing proteins in the periplasm, Amp^R^, T7 promoter	Laboratory stock
pET39b	Encodes Dsb tag for export and periplasmic folding of target proteins, Km^R^, T7 promoter	Laboratory stock
pET43.1a	High-level expression of peptide sequences fused with the 491 aa Nus•Tag™ protein, Amp^R^, T7 promoter	Laboratory stock
pET48b	Encodes Trx tag, Km^R^, T7 promoter	Laboratory stock
pMD18T-B_74_A	pMD18T, *p4h*-*proB*_*74*_-*proA*	Laboratory stock
LGOX	pET28a, *lgox*	Laboratory stock
p1	pET24a, P*trp*::*p4h*-*proB*_*74*_-*proA*	This study
p2	pET24a, P*trp*::*p4h*	This study
p3	pET24a, P*trp*::*p4h*-*proB*_*74*_	This study
p4	pET24a, P*trp*::*p4h*-*proB*_*74*_-*proA*-*proC*	This study
p5	pACYC-Duet-1, P*trp*::*p4h*	This study
p6	pET24a, *p4h*	This study
p7	pKK223, *p4h*	This study
p8	pGEX-6P-1, *p4h*	This study
p9	pMAL-C2-X, *p4h*	This study
p10	pET20b, *p4h*	This study
p11	pET39b, *p4h*	This study
p12	pET43.1a, *p4h*	This study
p13	pET48b, *p4h*	This study
p14	pMAL-C2-X, P*tac*::MBP-*p4h*-*proB*_*74*_-*proA*-*proC*	This study
p15 (H0)	pET24a, P*trp*::MBP-*p4h*-*proB*_*74*_-*proA*-*proC*	This study
p16	pACYC-Duet-1, P*trp*::MBP-*p4h*	This study
p17	pACYC-Duet-1, P*tac*::MBP-*p4h*	This study
p18	pACYC-Duet-1, P*trp*::*proB*_*74*_-*proA*-*proC*	This study
p19	pACYC-Duet-1, P*tac*::*proB*_*74*_-*proA*-*proC*	This study
H1	pET24a, P*trp*::MBP-*p4h*-P*trp*::*proB*_*74*_-*proA*-*proC*	This study
H2	pET24a, P*trp*::MBP-*p4h*-P*tac*::*proB*_*74*_-*proA*-*proC*	This study
H3	pET24a, P*tac*::MBP-*p4h*-*proB*_*74*_-*proA*-*proC*	This study
H4	pET24a, P*tac*::MBP-*p4h*-P*trp*::*proB*_*74*_-*proA*-*proC*	This study
H5	pET24a, P*tac*::MBP-*p4h*-T7 t-P*trp*::*proB*_*74*_-*proA*-*proC*	This study
H6	pET24a, P*tac*::MBP-*p4h*-P*tac*::*proB*_*74*_-*proA*-*proC*	This study
H7	pET24a, P*tac*::MBP-*p4h*-T7 t-P*tac*::*proB*_*74*_-*proA*-*proC*	This study
p20	pACYC-Duet-1, P*trp*:: *lgox*	This study
p21	pACYC-Duet-1, P*tac*:: *lgox*	This study
p22	pACYC-Duet-1, P*trp*::*icd*-*gltA*-*acs*	This study
p23	pACYC-Duet-1, P*tac*::*icd*-*gltA*-*acs*	This study
K0	pACYC-Duet-1, Ptrp::*lgox*-*icd*-*gltA*-*acs*	This study
K1	pACYC-Duet-1, P*trp*::*lgox*-P*trp*::*icd*-*gltA*-*acs*	This study
K2	pACYC-Duet-1, P*trp*::*lgox*-P*tac*::*icd*-*gltA*-*acs*	This study
K3	pACYC-Duet-1, P*tac*::*lgox*-*icd*-*gltA*-*acs*	This study
K4	pACYC-Duet-1, P*tac*::*lgox*-P*trp*::*icd*-*gltA*-*acs*	This study
K5	pACYC-Duet-1, P*tac*::*lgox*-T7 t-P*trp*::*icd*-*gltA*-*acs*	This study
K6	pACYC-Duet-1, P*tac*::*lgox*-P*tac*::*icd*-*gltA*-*acs*	This study
K7	pACYC-Duet-1, P*tac*::*lgox*-T7 t-P*tac*::*icd*-*gltA*-*acs*	This study
p24	pET24a, P*trp*::MBP-*p4h*-*proB*_*74*_*-proA*-*proC*-*lgox*	This study

The codon-optimized gene for *p4h* from *Dactylosporangium* sp. RH1 was synthesized and introduced into a pET24a plasmid to generate the p6 plasmid. Based on the p6 plasmid, the p2 vector was constructed by replacing the T7 promoter with the trp promoter. The *proB*_*74*_ and *proA* genes (from *E. coli* W3110) were PCR amplified from pMD18T-B_74_A containing the *proB* mutant gene and *proA* gene and were then introduced into a p2 plasmid to generate p1. Similarly, p3 and p4 were obtained, and *proC* genes were obtained from *E. coli* W3110 genomic DNA.

To improve the expression of P4H, the *p4h* gene was introduced into pKK223-3, pGEX-6P-1, pMAL-C2-X, pET20b, pET39b, pET43.1a, and pET48b plasmids to generate p7, p8, p9, p10, p11, p12, and p13, respectively.

The sequence containing the trp promoter with the *p4h* gene was amplified from p2 and ligated into pACYC-Duet-1 to obtain p5. The *p4h*, *proB*_*74*_, *proA*, and *proC* genes were amplified from the p4 plasmid and ligated into pMAL-C2-X with a one-step cloning kit to obtain p14. The *malE* gene was amplified from the plasmid pMAL-C2-X and ligated into p4 to obtain p15 (H0). The *MH* (MBP-*p4h*) genes were amplified from the p15 (H0) plasmid and ligated into p5, creating p16. The fragment containing the tac promoter, RBS, MCS, and the terminator from pKK223-3 was ligated into pACYC-Duet-1, creating pACYC-tac. Then, the *MH* genes were ligated into pACYC-tac, creating p17. The *B*_*74*_*AC* (*proB*_*74*_, *proA*, and *proC*) genes were ligated into p5, creating p18. The *B*_*74*_*AC* genes were also ligated into pACYC-tac, creating p19. To introduce a heterologous α-KG biosynthesis pathway**,** gene* L* (*lgox*) was amplified from the LGOX plasmid. To increase the production of the endogenic α-KG biosynthesis pathway, *IGa* (*icd*, *gltA*, and *acs*) genes were amplified from *E. coli* W3110 genomic DNA. Then, the plasmids p20, p21, p22, and p23 were constructed using a similar method.

For construction of the plasmids harboring H modules, a sequence with the trp promoter and *B*_*74*_*AC* were ligated into pEtrp-MH to obtain H1 by Gibson assembly. H2-H7 plasmids were constructed using the same method. For construction of plasmids harboring K modules, *IGa* genes were ligated into p20 to obtain K0 and, similarly, K1-K7 plasmids were constructed using the Gibson assembly. Finally, the *L* gene was ligated into p15 (H0) to obtain p24.

### Gene deletions in *Escherichia coli* W3110

Genes in *E. coli* W3110 were deleted using a Red/ET recombination system [37]. The deletion cassettes were prepared by PCR amplification, and pKD46 was transformed into *E. coli* W3110. Then, the deletion cassettes were integrated into the chromosome of *E. coli* W3110. The resistance genes were removed by introducing pCP20, which carried the gene encoding FLP recombinase.

### Determination of proline-4-hydroxylase activity levels

P4H activity levels were measured through whole-cell reaction procedures. After 8 h of induction in fermentation medium, cell optical density was measured at 600 nm using a spectrophotometer and, according to the proper formula, cell dry weight was calculated. The cells were harvested by centrifugation at 12,000 × g for 5 min. The harvested cells were resuspended in500 μL of reaction mixture (240 mM pH 6.5 2-[N-morpholino] ethanesulfonic acid (MES) buffer, 20 mM L-proline, 40 mM α-KG, 4 mM FeSO_4_, and 8 mM ascorbate). The reaction mixture samples were incubated at 35 °C for 15 min, and then, cellular activity was terminated completely by heat treatment at 100 °C for 2 min. The amount of *t*4Hyp in the supernatant of each sample was determined after centrifugation. The amount of enzyme that formed 1 nmol of *t*4Hyp in one minute is defined as 1 U Whole-cell enzyme activity (U g^−1^) indicated the enzymatic activity per gram of cell dry weight.

### Analytical methods

The cell concentration was determined based on the OD measured at 600 nm using a spectrophotometer (Shunyuhengping, Shanghai, China), and one OD unit corresponded to 0.375 g/L of cell dry weight [38]. Quantification of proline and *t*4Hyp was performed on the basis of high-performance liquid chromatography (Model 1100, Agilent, Santa Clara, USA) with a system equipped with a UV detector. Analytes were separated on a C18 column (RP18 5 μm 4.6 × 250 mm, Waters, USA) maintained at 35 °C. Glucose was quantified with a SBA biosensor. α-KG was measured by high-performance liquid chromatography with a Sugar-H column eluted with 5 mM H_2_SO_4_ at a flow rate of 0.6 mL/min and 50 °C.

## Supplementary Information

**Additional file 1: Table S1. **Primers and their sequences used for PCR in this study. **Table S2. **Mobile phase gradient for separation of proline and *trans*-4-hydroxy-L-proline via HPLC.

## Data Availability

All the data generated or analyzed during this study are included in published article and its additional file.

## Abbreviations

*t*4Hyp: *trans-*4-Hydroxy-L-proline
*E. coli*: *Escherichia coli*
TCA cycle: Tricarboxylic acid cycle
IPTG: Isopropyl β-d-thiogalactoside
RBS: Ribosome binding site
MCS: Multiple cloning site
α-KG: α-Ketoglutarate
MSG: Monosodium glutamate
