# Supplementary material for: Modular reconstruction and optimization of the trans-4-hydroxy-L-proline synthesis pathway in Escherichia coli
Source: Microb Cell Fact. 2022 Aug 11;21:159. doi: 10.1186/s12934-022-01884-4 (PMC9367115; doi:10.1186/s12934-022-01884-4)
Supplement: Supplementary file 1 — Additional file 1: Table S1. Primers and their sequences used for PCR in this study. Table S2. Mobile phase gradient for separation of proline and trans-4-hydroxy-L-proline via HPLC. [file 12934_2022_1884_MOESM1_ESM.docx]

**Table S1** Primers and their sequences used for PCR in this study.

| Names | 5’-3’ sequences | Restriction sites | |
| --- | --- | --- | --- |
| F-proBA-*Bam*HI | CATGGATCCAGAGAATCATGAGTGACAGCC | *Bam*HI |  |
| R-proBA-*Hind*III | GCTAAGCTTCGTCAATGGCCTTGTGAATC | *Hind*III |  |
| F-putA | GGAGGTTGTAACATCCTCCGGCTACCTGTTTAACCTATAGTCATTAAGCTATTCCGGGGATCCGTCGACC |  |  |
| R-putA | TCACGATAACGTTAAGTTGCACCTTTCTGAACAACAGGAGTAATGGCATGTGTAGGCTGGAGCTGCTTCG |  |  |
| F-sucA | AGTATCCACGGCGAAGTAAGCATAAAAAAGATGCTTAAGGGATCACGATGATTCCGGGGATCCGTCGACC |  |  |
| R-sucA | ATCTACGCTACTCATTGTGTATCCTTTATTTATTCGACGTTCAGCGCGTCTGTAGGCTGGAGCTGCTTCG |  |  |
| F-aceA | TACCGCCTGTTAGCGTAAACCACCACATAACTATGGAGCATCTGCACATGATTCCGGGGATCCGTCGACC |  |  |
| R-aceA | GGCCTACAGTCAGCAACGGTTGTTGTTGCTTAGAACTGCGATTCTTCAGTTGTAGGCTGGAGCTGCTTCG |  |  |
| F-sucCD | GGTCTACGGTTTAAAAGATAACGATTACTGAAGGATGGACAGAACACATGGATCCGTCGACCTGCAGTTC |  |  |
| R-sucCD | CGGCGAGGGCTATTTCTTATTACAGATATTTATTTCAGAACAGTTTTCAGGTGTAGGCTGGAGCTGCTTC |  |  |
| F-proB-*Bam*HI | CATGGATCCAGAGAATCATGAGTGACAGCC | *Bam*HI |  |
| R-proB-*Eco*RI | CCGGAATTCTTAACGGGTAATCATGTCAT | *Eco*RI |  |
| F-proC-*Hind*III | GCTAAGCTTCGGCAGGAGTGAGGCAATGG | *Hind*III |  |
| R-proC-*Not*I | ATTTGCGGCCGCCGTCCGGCGAAAGTCATC | *Not*I |  |
| F-P4H-*Bam*HI | CATGGATCCATGCTGACCCCGACCG | *Bam*HI |  |
| R-P4H-*Hind*III-*Xho*I | GCTAAGCTTCTCGAGTTAAACCGGCT | *Hind*III, *Xho*I |  |
| F-trpH-*Bam*HI | CATGGATCCGGTCCGGCGTAGAGGATCGAG | *Bam*HI |  |
| R-trpH-*Hind*III | GCTAAGCTTTTAAACCGGCTGAGCCAGAGC | *Hind*III |  |
| F-HBAC | GAGGGAAGGATTTCAGAATTCATGCTGACCCCGACCGAACTG | *Eco*RI |  |
| R-HBAC | TGCCTGCAGGTCGACGAATTCTCAGGATTTGCTGAGTTTTTCTG | *Eco*RI |  |
| F-MBP-*Nde*I | CACGTAAAAAGGGTATCGATCATATGAAAATCGAAGAAGGTAAACTG | *Nde*I |  |
| R-MBP-*Nde*I | CAGTTCGGTCGGGGTCAGCATATGAGTCTGCGCGTCTTTCAGGG | *Nde*I |  |
| F-P13 | AAAAAGGGTATCGATCATATGAAAATCGAAGAAGGTAAACTGG | *Nde*I |  |
| R-13 | CCAATTGAGATCTGCCATATGTTAAACCGGCTGAGCCAGAG | *Bgl*II, *Nde*I |  |
| F-P14 | AAAAAGGGTATCGATCATATGAACGAGATGACCTACGAGCA | *Nde*I |  |
| R-P14 | CCAATTGAGATCTGCCATATGTCATGACGTCAGTGCTTCCTCTC | *Bgl*II, *Nde*I |  |
| F-P15 | AAAAAGGGTATCGATCATATGAGTGACAGCCAGACGCTG | *Nde*I |  |
| R-P15 | CCAATTGAGATCTGCCATATGTCAGGATTTGCTGAGTTTTTCTGA | *Bgl*II, *Nde*I |  |
| F-P16I | AAAAAGGGTATCGATCATATGGAAAGTAAAGTAGTTGTTCCGG | *Nde*I |  |
| R-P16I | CAGCCATTTACATGTTTTCGATGATCGCG |  |  |
| F-P1619G2 | CGATCATCGAAAACATGTAAAGGAGTATGGCTGATACAAAAGCAAA |  |  |
| R-P1619G2 | TGTTTGTGAATTTGGCTCATACTCCTTTAACGCTTGATATCGCTTT |  |  |
| F-P1619a2 | AAAGCGATATCAAGCGTTAAAGGAGTATGAGCCAAATTCACAAACA |  |  |
| R-P16a | CCAATTGAGATCTGCCATATGTTACGATGGCATCGCGATAGC | *Bgl*II, *Nde*I |  |
| F-P17 | TTCACACAGGAAACAGAATTCATGAAAATCGAAGAAGGTAAACTGG | *EcoR*I |  |
| R-P17 | CATCCGCCAAAACAGAAGCTTTTAAACCGGCTGAGCCAGAG | *Hind*III |  |
| F-P18 | TTCACACAGGAAACAGAATTCATGAGTGACAGCCAGACGCTG | *EcoR*I |  |
| R-P18 | CATCCGCCAAAACAGAAGCTTTCAGGATTTGCTGAGTTTTTCTGA | *Hind*III |  |
| R-P1619I | TTTGCTTTTGTATCAGCCATACTCCT TTACATGTTTTCGATGATCG |  |  |
| R-P19a | CATCCGCCAAAACAGAAGCTTTTACGATGGCATCGCGATAGC | *Hind*III |  |
| F-H1 | GCTCAGCCGGTTTAAGGATCCGGTCCGGCGTAGAGGATCG | *BamH*I |  |
| R-H124567 | TGGTGGTGCTCGAGTGCGGCCGCTCAGGATTTGCTGAGTTTTTCTGA | *Not*I |  |
| F-H2 | GCTCAGCCGGTTTAAGGATCCGTAATTGACAATTAATCATCGGCTCG | *BamH*I |  |
| F-H34567 | GGCGTAGAGGATCGAGATCCGTAATTGACAATTAATCATC |  |  |
| R-H3 | CACTCATGATTCTCTGGATCCTTAAACCGGCTGAGCCAGAG | *BamH*I |  |
| R-H4-MH | TCGATCCTCTACGCCGGACCTTAAACCGGCTGAGCCAGAG |  |  |
| F-H4-BAC | CTCTGGCTCAGCCGGTTTAAGGTCCGGCGTAGAGGATC |  |  |
| R-H5-MH | TCGATCCTCTACGCCGGACCAAAAGGCCATCCGTCAGGAT |  |  |
| F-H5-BAC | ATCCTGACGGATGGCCTTTTGGTCCGGCGTAGAGGATC |  |  |
| R-H6-MH | GATGATTAATTGTCAATTACTTAAACCGGCTGAGCCAGAG |  |  |
| F-H6-BAC | CTCTGGCTCAGCCGGTTTAAGTAATTGACAATTAATCATC |  |  |
| R-H7-MH | GATGATTAATTGTCAATTACAAAAGGCCATCCGTCAGGAT |  |  |
| F-H7-BAC | ATCCTGACGGATGGCCTTTTGTAATTGACAATTAATCATC |  |  |
| F-K0-R | GTCATGACATATGGCAGATCTAGGAGTATGGAAAGTAAAGTAGTTGTTCCGG | *Nde*I, *Bgl*II |  |
| R-K012 | GCCGATATCCAATTGAGATCTTTACGATGGCATCGCGATAGC | *Bgl*II |  |
| F-K1 | GTCATGACATATGGCAGATCTGGTCCGGCGTAGAGGATCG | *Nde*I, *Bgl*II |  |
| F-K2 | GTCATGACATATGGCAGATCTGTAATTGACAATTAATCATCGGCTCG | *Nde*I, *Bgl*II |  |
| F-K3-R | GCACTGACGTCATGAAAGCTTAGGAGTATGGAAAGTAAAGTAGTTGTTCCGG | *Hind*III |  |
| R-K346 | CATCCGCCAAAACAGAAGCTTTTACGATGGCATCGCGATAGC | *Hind*III |  |
| F-K4 | GCACTGACGTCATGAAAGCTTGGTCCGGCGTAGAGGATCG | *Hind*III |  |
| F-K5 | AGATATACATATGGCAGATCTGGTCCGGCGTAGAGGATCG | *Nde*I, *Bgl*II |  |
| R-K57 | GCCGATATCCAATTGAGATCTTTACGATGGCATCGCGATAGC | *Bgl*II |  |
| F-K6 | GCACTGACGTCATGAAAGCTTGTAATTGACAATTAATCATCGGCTCG | *Hind*III |  |
| F-K7 | AGATATACATATGGCAGATCTGTAATTGACAATTAATCATCGGCTCG | *Nde*I, *Bgl*II |  |

**Table S2** Mobile phase gradient for separation of proline and *trans*-4-hydroxy-L-proline via HPLC.

| Time (min) | Mobile phase A (%) | Mobile phase B (%) |
| --- | --- | --- |
| 0.0 | 0 | 100 |
| 12.0 | 57.5 | 42.5 |
| 13.1 | 100 | 0 |
| 13.5 | 100 | 0 |
| 18.9 | 100 | 0 |
| 19.0 | 0 | 0 |
| 20.0 | 0 | 100 |
| 22.0 | 0 | 100 |

Mobile phase A: 0.02 mol/L sodium acetate: acetonitrile: methyl alcohol=1:2:2; Mobile phase B: 0.02 mol/L sodium acetate.
